# 

**Published:** 1878-04

**Authors:** 


### Books and Pamphlets Received.

---

Injuries of the Eye, and their Medico-Legal aspect, by Ferdinand Von Arlt, M. D. Translated by Chas. S. Turnbull, M. D. Philadelphia: Claxton, Remsen and Haffelfinger. 1878.

The Source of Muscular Power, by Austin Flint, Jr., M. D. New York: D. Appleton & Co. 1878.

Reference and Dose Book, by C. Henri Leonard, M. A., M. D. Third Edition. Detroit. 1878. Price, 75 cents. pp. 100

Report of Committee on Military affairs. Bill for the relief of Wm. A. Hammond, M. D.

The action of the Nervous System, by John J. Caldwell, M. D. Baltimore. 1877.

Bathing, Cupping, Electricity, Massage, by David Prince, M. D. Reprint from *American Practitioner.* February, 1878.

Hydrobromic Acid, by Edward R. Squibb, M. D.

Operative Treatment of Vascular Tumors of the Eyelids, by H. Knapp.

On the operations for Traumatic Colobomata of the Eyelids, by H. Knapp. Reprint from Archives of Ophthalmology and Otology. Vol. V. No. 1. 1876.

Meteorology in the Service of Medicine, by Dr. J. Schrieber. Translated by W. H. Sedding, M. D. *Richmond and Louisville Medical Journal.* Feb. 1878.

Report on Dependent and Delinquent children, by Wm. P. Letchworth, of Buffalo, N. Y.

Twenty-fifth Annual Report of the Penn. Training School for Feeble-minded Children.

Proceedings of the Association of Medical Officers of American Institutions for Idiotic and Feeble-minded persons. 1876 and 1877.

Report of Penn. Hospital for the Insane for the year 1877.

Report of the Butler Hosp. for the Insane. January, 1878.

Annual announcement of the Med. College of the Pacific, session of 1878.

Higher Medical Education, the true interest of the public and of the profession, by Wm. Pepper, A. M., M. D. Philadelphia. 1877.

Lectures and Essays, by Virgil W. Blanchard, M. D. New York. 1877.

The Truth Admitted. The Columbia Hosp. for Women at Washington. Reprint from Richmond and Louisville Med. Jour, Jan. 1878.

### Contents of April Number.

### ORIGINAL.

**THE BUFFALO**

**Medical and Surgical Journal**

**PUBLISHED MONTHLY,**

Containing Original Articles, Reports of Medical Societies and Hospitals, Editorial, Reviews, Correspondence, Army News, etc., etc ; including the usual variety of Medical Periodical Publications. Specimen copies sent on application.

Address Buffalo Medical & Surgical Journal, Buffalo, N. Y.

TERMS OF SUBSCRIPTION, $3.00 PER YEAR, IN ADVANCE.

All communications for publication shoud be sent before the fifteenth (15th) of each month, or they will of necessity lie over until the next number. No change can be made in advertisements after the twenty-fifth. Address, Buffalo Medical and Surgical Journal, 978 Main Street.

Correspondence and original articles are solicited. Publication is no evidence that the views of the author are endorsed.

